# New-Onset Graves’ Ophthalmopathy After Treatment with Pembrolizumab: A Case Report and a Review of the Literature

**DOI:** 10.3390/diagnostics15212764

**Published:** 2025-10-31

**Authors:** Moduo Pan, Xuecong Zhou, Yuan Wu

**Affiliations:** Department of Ophthalmology, Peking University First Hospital, No. 8 Xi Shi Ku Street, Xi Cheng District, Beijing 100034, China; panmoduo@163.com (M.P.); zhouxuecong1128@163.com (X.Z.)

**Keywords:** Graves’ ophthalmopathy, Graves’ disease, pembrolizumab, immune checkpoint inhibitors, immune-related adverse events

## Abstract

**Background and Clinical Significance:** Immune checkpoint inhibitors (ICIs), a revolutionary class of oncology therapeutics that enhance T cell-mediated antitumor immunity, are associated with various immune-related adverse events (IRAEs). While destructive thyroiditis and hypothyroidism are common, ICI-induced Graves’ disease (GD) is exceedingly rare, and the occurrence of concomitant Graves’ ophthalmopathy (GO) is even rarer. **Case Presentation:** A 57-year-old man with bladder cancer developed GO after receiving the first dose of the programmed death 1 (PD-1) inhibitor pembrolizumab. He presented with severe proptosis, extraocular muscle enlargement, hyperthyroidism, and significantly increased thyroid-stimulating hormone receptor autoantibodies (TRAb). Following the treatment with glucocorticoids and immunosuppressive therapy, his symptoms improved markedly but relapsed upon dosage reduction. To date, we have not identified any previous reports of GO with confirmed positive thyroid-related antibodies induced by pembrolizumab. **Conclusions:** This case offers valuable insights into the potential IRAEs, underscoring the importance of thorough clinical evaluation and early recognition to improve patient outcomes and quality of life. A literature review of ICI-induced GO was also performed, with further discussion of the potential pathogenic mechanisms, risk factors, and management strategies.

## 1. Introduction

Immune checkpoint inhibitors (ICIs) are revolutionizing therapy in oncology, with promising results in various types of cancer. By inhibiting the interaction between immune checkpoints and their ligands, ICIs disable the ability of cancer cells to evade the immune system surveillance mechanisms, restoring T cell-mediated antitumor immunity [1].

Pembrolizumab (Keytruda), a humanized monoclonal antibody against programmed death 1 (PD-1), has been approved by the US Food and Drug Administration (FDA) for use in advanced melanoma, non-small-cell lung carcinoma, head and neck squamous cell carcinoma, urothelial carcinoma, and classic Hodgkin lymphoma [2].

Like other ICIs, pembrolizumab can cause immune-related adverse events (IRAEs) by blocking negative regulators of adaptive immunity in the endocrine, rheumatological, gastrointestinal, pulmonary, cardiovascular, and neurological systems [3]. Although thyroid dysfunction is one of the most common endocrine IRAEs of pembrolizumab [4,5], the mechanism usually involves destructive thyroiditis rather than Graves’ disease (GD) [6]. Graves’ ophthalmopathy (GO) is even rarer and has seldom been reported. Here, we present a case of GO that occurred shortly after initiating pembrolizumab therapy for bladder cancer.

## 2. Case Report

This is a 57-year-old man who was diagnosed with invasive urothelial carcinoma of the bladder in 2017 and underwent partial cystectomy, but experienced a recurrence six years later. After undergoing 28 sessions of radiation therapy and 4 cycles of gemcitabine chemotherapy, he started treatment with the PD-1 inhibitor, pembrolizumab.

After the first dose of pembrolizumab, he developed palpitations, dyspnea, and fever. His thyroid function tests revealed hyperthyroidism with elevated levels of triiodothyronine (T3) (6.95 nmol/L; normal range 0.92–2.79 nmol/L), free triiodothyronine (FT3) (>30.80 pmol/L; normal range 3.50–6.50 pmol/L), thyroxine (T4) (>387.00 nmol/L; normal range 58.1–140.6 nmol/L), and free thyroxine (FT4) (117.70 pmol/L; normal range 11.48–22.70 pmol/L). Thyroid-stimulating hormone (TSH) was decreased (0.01 μIU/mL; normal range 0.55–4.78 μIU/mL). Notably, his thyroid-stimulating hormone receptor autoantibodies (TRAb) (>40.00 IU/L; normal range 0–1.75 IU/L) and thyroid-stimulating immunoglobulin (TSI) (23.20 IU/L; normal range <0.55 IU/L) levels significantly increased (Table 1). Thyroid ultrasonography revealed that both thyroid lobes were 2.0 cm thick, with a heterogeneous echotexture.

**Figure 1:**
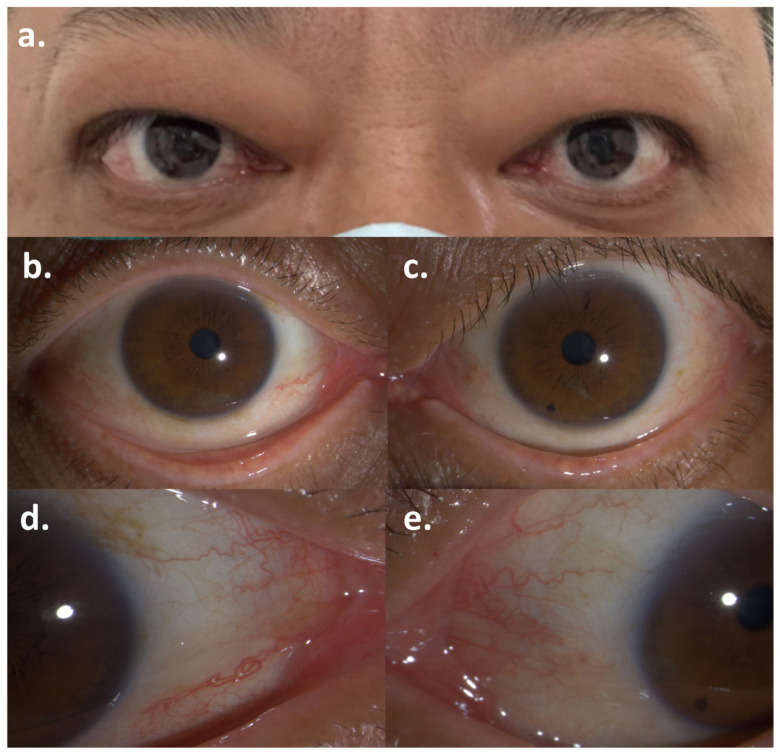
Symptoms of Graves’ ophthalmopathy after the first dose of Pembrolizumab: (**a**) Bilateral exophthalmos; (**b**,**c**) Conjunctival injection of the right eye and the left eye; (**d**,**e**) Caruncular congestion of the right eye and the left eye.

**Figure 2:**
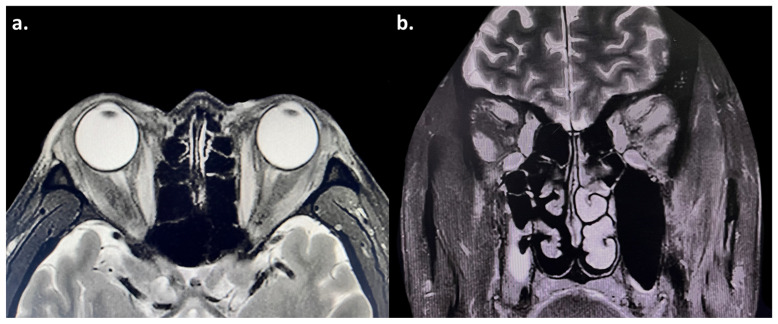
(**a**,**b**) Magnetic resonance imaging (MRI) showed enlargement of the bilateral medial rectus, superior rectus, superior oblique, inferior rectus, and lateral rectus muscles, with hyperintensity on T2-weighted images. The bilateral optic nerves were normal.

Soon after, he developed bilateral exophthalmos, measuring 21 mm in the right eye and 20 mm in the left (Table 2). He also had eyelid swelling and erythema, conjunctival injection, caruncular congestion and swelling (Figure 1), ocular pain, mild diplopia, and restricted eye movements. On ophthalmic examination, he had visual acuity of 20/20 bilaterally and intraocular pressure of 25 mmHg bilaterally. Orbital magnetic resonance imaging (MRI) showed enlargement of bilateral medial rectus, superior rectus, superior oblique, inferior rectus, and lateral rectus muscles, with hyperintensity on T2-weighted images (Figure 2). He was diagnosed with moderate-to-severe active GO with a clinical activity score (CAS) of 5/7 points (retrobulbar pain, redness of eyelids, redness of conjunctiva, swelling of caruncle, swelling of eyelids), attributed to ICI therapy. Although intravenous glucocorticoid pulse therapy is typically recommended as first-line treatment, it was considered with caution due to the patient’s oncological background. High-dose systemic glucocorticoids carry a risk of broad immunosuppression, which could potentially compromise cancer control and lead to disease progression. Therefore, the patient was administered a single retrobulbar injection of 20 mg compound betamethasone. Concurrently, oral medication was initiated with mycophenolate mofetil (0.5 g twice daily for 3 months) along with prednisolone acetate (30 mg once daily), which was tapered over one month. Methimazole (10 mg once daily) was also prescribed for the management of hyperthyroidism.

**Figure 3:**
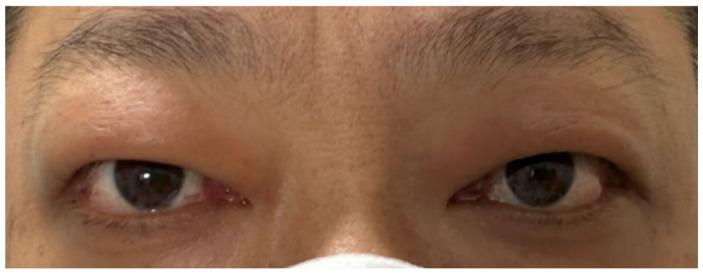
Symptoms of Graves’ ophthalmopathy had improved.

After 3 months of treatment, the patient had notable improvement: the conjunctival and caruncular congestion subsided, ocular pain vanished, and diplopia disappeared, resulting in a CAS of 2/7 points (redness of eyelids, swelling of eyelids) (Figure 3). Given the marked improvement and the need to avoid prolonged immunosuppressive therapy in patients with malignancies, the dosage of mycophenolate mofetil was reduced by half to 0.25 g twice daily. The methimazole dosage was maintained at 10 mg once daily.

**Figure 4:**
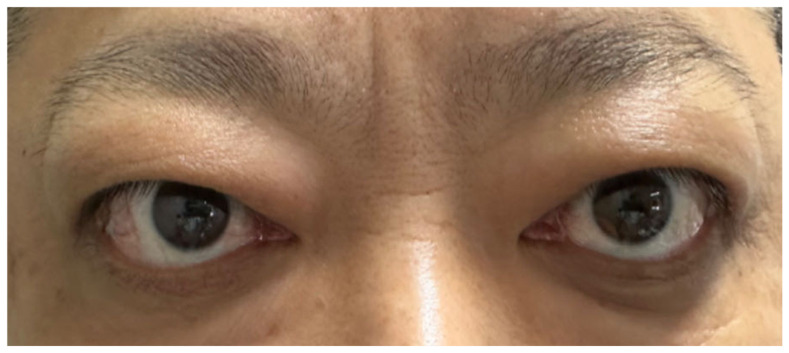
Symptoms of Graves’ ophthalmopathy had relapsed.

**Figure 5:**
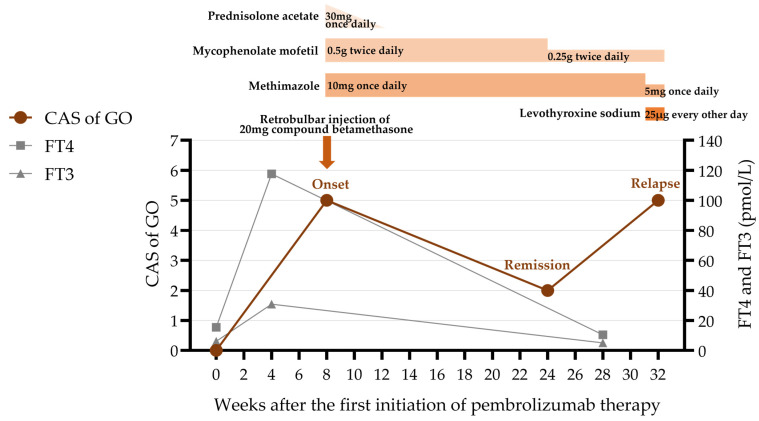
The clinical course including the patient’s symptoms, medication administered, and thyroid function. CAS = clinical activity score; GO = Graves’ ophthalmopathy; FT4 = free thyroxine; FT3 = free triiodothyronine.

Later, the patient’s GO relapsed, with exophthalmos measuring 23 mm in the right eye and 25 mm in the left. Levels of TRAb (6.38 IU/L; normal range 0–1.75 IU/L) and TSI (3.10 IU/L; normal range <0.55 IU/L) increased again. Subsequently, symptoms of conjunctival and lacrimal gland congestion, ocular pain, diplopia, and limited ocular motility recurred (Figure 4), pushing the CAS to 5/7 points again (retrobulbar pain, redness of eyelids, redness of conjunctiva, swelling of caruncle, swelling of eyelids) (Figure 5). Notably, the hyperthyroidism did not recur concurrently with GO; instead, the patient developed hypothyroidism, characterized by decreased FT4 (10.41 pmol/L; normal range 11.48–22.70 pmol/L) and elevated TSH (9.05 μIU/mL; normal range 0.55–4.78 μIU/mL). We therefore introduced levothyroxine sodium (25 μg every other day) to correct the hypothyroidism. As methimazole provides immunomodulatory benefits and helps lower antibody levels—which remained elevated—it was not discontinued but maintained at a reduced dose (5 mg once daily).

## 3. Discussion

Immune checkpoint inhibitors represent a revolutionary class of tumor immunotherapy drugs. By blocking intrinsic downregulators of immunity, such as cytotoxic T-lymphocyte antigen 4 (CTLA-4), programmed death 1 (PD-1), and programmed death-ligand 1 (PD-L1), they disrupt the immune escape mechanism of tumor cells and significantly enhance anti-tumor immune responses.

CTLA-4 (CD152) is a T-cell surface receptor belonging to the B7/CD28 immunoglobulin superfamily. It mediates immunosuppression by negatively regulating T-cell activation through inhibition of CD28-mediated costimulatory signals [2]. Ipilimumab was the first CTLA-4 inhibitor approved by the FDA in 2011 for the treatment of melanoma. Another target of immune checkpoint inhibitors, PD-1, is a type I transmembrane protein widely expressed on T/B cells, natural killer cells, and so on. Binding to its ligands PD-L1 and PD-L2 enables PD-1 to inhibit tumor cell apoptosis and promote the development of peripheral T-effector cell exhaustion [7]. Nivolumab, the first PD-1-targeting human IgG4 monoclonal antibody, was approved by the FDA in 2014. It was initially for advanced melanoma treatment, with subsequent indications expanded to non-small cell lung cancer, renal cell carcinoma, hepatocellular carcinoma, classic Hodgkin’s lymphoma, head and neck squamous cell carcinoma, urothelial carcinoma, and so on [3]. Pembrolizumab also obtained FDA approval in 2014, first for advanced melanoma and later for non-small cell lung cancer, classical Hodgkin lymphoma, head and neck squamous cell carcinoma, and urothelial carcinoma [8].

As ICIs broadly activate the immune system by relieving T-cell inhibitory signals, they may cause multi-system immune-related adverse events. While thyroid-related adverse events are rather common, they typically manifest as destructive thyroiditis rather than Graves’ disease [6]. GD is an autoimmune disease caused by the stimulatory activity of TRAb, presenting with symptoms of thyrotoxicosis and/or orbitopathy. The mechanism underlying PD-1 inhibitor-induced GD remains incompletely understood. A possible mechanism is that PD-1 inhibitors such as pembrolizumab block the interaction between PD-1 and its ligands, which plays a critical role in down-regulating immune responses and preventing autoimmune disorders. This blockade leads to autoimmune system activation, thereby inducing the occurrence of GD [9].

Among patients receiving ICIs therapy, the incidence of ocular IRAEs is approximately 1% [10], mainly involving uveitis and conjunctivitis [11]. Graves’ ophthalmopathy, characterized by exophthalmos and limited ocular motility, is extremely rare. Our literature review identified only 9 documented cases of GO induced by ICI therapy (Table 3). Among these, anti-CTLA-4 was involved in 5 cases [12,13,14,15,16], mostly ipilimumab; anti-PD-1 was involved in 2 cases [17,18]; and a combination of anti-CTLA-4 and anti-PD-1/PD-L1 inhibitors was involved in 2 cases [16,19]. Min et al. [12] reported the first case of GO caused by anti-CTLA-4 therapy in 2010. A 51-year-old woman with stage IV melanoma experienced acute ocular pain, conjunctivitis, bilateral proptosis, and periorbital edema after four doses of ipilimumab at 10 mg/kg. MRI showed bilateral thickening of the extraocular muscles. Laboratory tests revealed normal TSH and FT4 levels; however, thyroid peroxidase antibody (TPOAb) and thyroglobulin antibody (TgAb) were significantly elevated. In 2018, Campredon et al. [17] reported the first case of orbital inflammation resembling thyroid eye disease caused by anti-PD-1 therapy, about a 61-year-old man with non-small cell lung cancer who developed severe proptosis, complete ophthalmoplegia, and conjunctival redness with chemosis after three nivolumab infusions. Orbital computed tomography (CT) showed marked bilateral proptosis and orbital fat expansion without significant extraocular muscle thickening. T2-weighted MRI displayed inflammatory changes similar to GO. Thyroid function tests indicated normal TSH and free T4 levels, with negative TRAb and TPOAb.

Notably, most cases presented as euthyroid GO [12,13,14,15,16,17,18,19]. Park et al. [18] described a 52-year-old man with recurrent metastatic Merkel cell carcinoma who developed progressive asymmetric proptosis, diplopia, and severe extraocular motility restriction after the third infusion of pembrolizumab. MRI revealed increased orbital fat volume, asymmetric proptosis, and mild tendon-sparing enlargement of the extraocular muscles. Due to normal T3/T4/TSH levels, he was diagnosed with euthyroid Graves’ ophthalmopathy, but without positive TRAb. Chang et al. [20] emphasized that a definitive diagnosis of GO relies on typical symptoms, positive thyroid antibodies, and supportive imaging findings. Here, we present a rare case of Graves’ ophthalmopathy with significant elevation of thyroid-related antibodies shortly after the pembrolizumab therapy for bladder cancer, which contributes to our understanding of IRAEs induced by ICIs.

The pathological features of GO include inflammation and infiltration of orbital tissues caused by immune cells, as well as the heterogeneity of fibroblasts and the production of extracellular matrix, ultimately leading to proptosis and restricted ocular motility [21]. The pathophysiology of GO is not fully known, while the breakdown of immune tolerance of thyroid-stimulating hormone receptor and insulin-like growth factor 1 receptor (IGF-1R) may play significant roles [22].

A personal history of thyroid disorders or autoimmune diseases may increase the risk of ICI-induced GO. Previously reported cases suggest that most GO occurs at least four weeks after the initiation of ICIs; however, Rhea et al. [15] reported an 83-year-old man with stage IIIC melanoma and a 17-year history of Graves’ disease. Within only three days of his first ipilimumab infusion (10 mg/kg), he developed new-onset moderate-to-severe GO featuring bilateral proptosis, chemosis, diplopia, and reduced visual acuity. Orbital CT demonstrated fusiform enlargement of the bilateral inferior rectus muscles. Although he maintained euthyroidism on levothyroxine, TRAb surged to 33.64 IU/L (normal range 0.00–1.75 IU/L). It is suggested that ICIs possibly induced the activation of previously existing cells that produce autoantibodies. The 2021 Society for Immunotherapy of Cancer clinical practice guidelines [23] reported that the risk of pre-existing autoimmune disease flare was 50% under ICI treatment. Therefore, in patients with pre-existing thyroid disorders or autoimmune diseases, ICI therapy requires more cautious administration and closer monitoring [24].

Glucocorticoids constitute the current first-line treatment for ICI-induced GO. Among previously reported cases, the majority of 8 patients treated with glucocorticoids exhibited symptom improvement or resolution, demonstrating significant efficacy. It should be noted that dose reduction or discontinuation carries a substantial risk of recurrence. For example, the patient with GO reported by Min et al. [12] resolved significantly after a course of oral prednisone, but relapse occurred two months later during prednisone dose reduction. Similarly, in our case, the patient’s GO improved markedly after the treatment with corticosteroids and immunosuppressants. However, a severe relapse occurred two months later after the dose reduction, which potentially led to the reemergence of immune responses directed against orbital tissues. With the gradual introduction of IGF-1R inhibitors such as teprotumumab, these agents may represent a preferred treatment option for this condition. However, at the time of this patient’s onset, IGF-1R inhibitors were not legally available in China.

Additionally, monitoring and maintaining normal thyroid function are also crucial for the management of GO. The development of hypothyroidism may also influence the recurrence [25], leading to an increase in TSH levels and stimulating the expression of thyroid-specific antigens [26], which in turn results in the reactivation of the inflammatory process. Sabini et al. [19] reported a 70-year-old man with advanced lung adenocarcinoma treated with tremelimumab and durvalumab. After one month of treatment, he developed GO accompanied by primary hypothyroidism. Laboratory analysis detected elevated TRAb, and orbital MRI confirmed enlargement of the inferior and medial rectus muscles, supporting a diagnosis of moderately severe, slightly active GO. Therefore, maintaining euthyroid status is essential for controlling the progression of GO, as both hyper- and hypothyroidism can have detrimental effects on it [27].

In conclusion, we reported a valuable case of GO induced by pembrolizumab, accompanied by hyperthyroidism and positive thyroid-associated antibodies, which has not been reported in previous cases. Following a systematic literature review of immune checkpoint inhibitor-associated GO, we analyzed its potential pathogenic mechanisms, risk factors, and management strategies. Although rare, GO can occur as an immune-related adverse event following ICI therapy and may present with variable thyroid function status. A personal history of thyroid or autoimmune disease may increase the risk of ICI-induced GO, underscoring the need for more cautious administration and closer monitoring in these patients. In terms of management, glucocorticoids remain the first-line treatment for ICI-induced GO; however, tapering should be gradual and closely monitored due to the substantial risk of recurrence. Additionally, maintaining euthyroidism is critical throughout the treatment, as both hyper- and hypothyroidism can exacerbate orbital disease activity. With the increasing availability of novel agents such as IGF-1R inhibitors, they may offer a promising alternative, particularly in cases where conventional immunosuppression is limited by oncology-related contraindications.

## Figures and Tables

**Table 1:** Results of thyroid function at different stages of Graves’ disease.

Parameter	Normal Range	Before Pembrolizumab Therapy		After Once Pembrolizumab Therapy		After 3 Months of Treatment	
		19 June 2023		13 July 2023		16 January 2024	
		Normal		Hyperthyroidism		Hypothyroidism	
T3 (nmol/L)	0.92–2.79	2.13	-	6.95	↑	1.84	-
FT3 (pmol/L)	3.50–6.50	6.11	-	>30.80	↑	5.06	-
T4 (nmol/L)	58.1–140.6	115.90	-	>387.00	↑	84.90	-
FT4 (pmol/L)	11.48–22.70	15.50	-	117.70	↑	10.41	↓
TSH (μIU/mL)	0.55–4.78	2.67	-	0.01	↓	9.05	↑
TRAb (IU/L)	0–1.75	na	na	>40.00	↑	6.38	↑
TSI (IU/L)	0–0.55	na	na	23.20	↑	3.10	↑

T3 = triiodothyronine; FT3 = free triiodothyronine; T4 = thyroxine; FT4 = free thyroxine; TSH = thyroid-stimulating hormone; TRAb = thyroid-stimulating hormone receptor autoantibodies; TSI = thyroid-stimulating immunoglobulin; na = not assessed; “↑” denotes above the normal range; “↓” denotes below the normal range; “-” denotes within the normal range.

**Table 2:** Results of ophthalmic examination at different stages of Graves’ ophthalmopathy.

	First Onset of GO		Remission of GO		Relapse of GO	
	August 2023		December 2023		February 2024	
	OD	OS	OD	OS	OD	OS
Retrobulbar pain	+	+	−	−	+	+
Pain with eye movement	−	−	−	−	−	−
Redness of eyelids	+	+	+	+	+	+
Redness of conjunctiva	+	+	−	−	+	+
Swelling of caruncle	+	+	−	−	+	+
Swelling of eyelids	+	+	+	+	+	+
Swelling of conjunctiva	−	−	−	−	−	−
Corneal epithelial damage	−	−	−	−	−	+
Protrusion	21-102-20		21-102-20		23-102-25	
MRD1	1	1	1	1	3	3
MRD2	5	5	5	5	8	8
CAS	5	5	2	2	5	5

GO = Graves’ ophthalmopathy; MRD1 = margin reflex distance-1; MRD2 = margin reflex distance-2; CAS = clinical activity score.

**Table 3:** Literature review: Comparison of case reports about ICI-induced Graves’ ophthalmopathy and thyroid eye disease-like orbital inflammation.

Case	ICI	Age/Sex	Tumor	Dosage	Time	History of the Thyroid Disease	Smoking	Thyroid Function	TRAb	TPOAb	Therapy	Outcome
**CTLA-4 Inhibitors**												
Min et al. 2010 [12]	Ipilimumab	51/F	Melanoma	10 mg/kg	12 w	No	na	Normal	Positive	Positive	Glucocorticoid	Symptoms resolved
Borodic et al. 2011 [13]	Ipilimumab	51/F	Melanoma	na	6 w	na	na	Normal	Positive	Positive	Cantholysis; Glucocorticoid	na
McElnea et al. 2014 [14]	Ipilimumab	68/F	Melanoma	3 mg/kg	6 w	No	na	Normal	Negative	Negative	Glucocorticoid	Partially improved
Rhea et al. 2018 [15]	Ipilimumab	83/M	Melanoma	10 mg/kg	3 d	Yes	na	Normal	Positive	na	Glucocorticoid	Symptoms resolved
Sagiv et al. 2019 [16]	Tremelimumab	51/M	Melanoma	10 mg/kg	24 w	na	na	Hyperthyroidism	Negative	Positive	Glucocorticoid	Symptoms resolved
**PD-1 Inhibitors**												
Campredon et al. 2018 [17]	Nivolumab	61/M	Non-small-cell lung cancer	na	6 w	No	Yes	Normal	Negative	Negative	Glucocorticoid	Partially improved
Park et al. 2018 [18]	Pembrolizumab	52/M	Merkel cell carcinoma	na	8 w	No	Yes	Normal	na	na	Glucocorticoid	Symptoms resolved
**CTLA-4 Inhibitors and PD-1/PD-L1 Inhibitors**												
Sabini et al. 2018 [19]	Tremelimumab+ Durvalumab	70/M	Lung adenocarcinoma	na	4 w	na	na	Hypothyroidism	Positive	na	Glucocorticoid	No response
Sagiv et al. 2019 [16]	Ipilimumab+ Nivolumab	73/M	Bladderurothelial carcinoma	1 mg/kg + 3 mg/kg	6 w	No	No	Normal	Negative	Negative	Glucocorticoid	Symptoms resolved

ICI = immune checkpoint inhibitors; TRAb = thyroid-stimulating hormone receptor autoantibodies; TPOAb = thyroid peroxidase antibody; CTLA-4 = cytotoxic T-lymphocyte antigen 4; PD-1 = programmed death 1; PD-L1 = programmed death-ligand 1; F = female; M = male; na = not assessed.

## Data Availability

The data presented in this study are available on request from the corresponding author. The data are not publicly available due to privacy and ethical reasons.

## Abbreviations

The following abbreviations are used in this manuscript:

ICIs	Immune checkpoint inhibitors
IRAEs	Immune-related adverse events
GD	Graves’ disease
GO	Graves’ ophthalmopathy
PD-1	Programmed death-1
TRAb	Thyroid-stimulating hormone receptor autoantibodies
FDA	Food and Drug Administration
T3	Triiodothyronine
FT3	Free triiodothyronine
T4	Thyroxine
FT4	Free thyroxine
TSH	Thyroid-stimulating hormone
TSI	Thyroid-stimulating immunoglobulin
MRI	Magnetic resonance imaging
CAS	Clinical activity score
MRD1	Margin reflex distance-1
MRD2	Margin reflex distance-2
CTLA-4	Cytotoxic T-lymphocyte antigen 4
PD-L1	Programmed death-ligand 1
TPOAb	Thyroid peroxidase antibody
TgAb	Thyroglobulin antibody
CT	Computed tomography
IGF-1R	Insulin-like growth factor 1 receptor
